# Covalent functionalization of polypropylene filters with diazirine–photosensitizer conjugates producing visible light driven virus inactivating materials﻿

**DOI:** 10.1038/s41598-021-98280-6

**Published:** 2021-09-24

**Authors:** T. J. Cuthbert, S. Ennis, S. F. Musolino, H. L. Buckley, M. Niikura, J. E. Wulff, C. Menon

**Affiliations:** 1 Department of Health Sciences and Technology, ETH Zürich, 8008 Zürich, Switzerland; 2 Schools of Mechatronic Systems Engineering and Engineering Science, Simon Fraser University, Metro Vancouver, BC V5A 1S6 Canada; 3 Faculty of Health Sciences, Simon Fraser University, Burnaby, BC V5A 1S6 Canada; 4 Department of Chemistry, University of Victoria, Victoria, BC V8W 3V6 Canada; 5 Department of Civil Engineering, University of Victoria, Victoria, BC V8W 3V6 Canada

**Keywords:** Materials for devices, Biomedical materials, Photochemistry, Polymer chemistry, Surface chemistry

## Abstract

The SARS-CoV-2 pandemic has highlighted the weaknesses of relying on single-use mask and respirator personal protective equipment (PPE) and the global supply chain that supports this market. There have been no major innovations in filter technology for PPE in the past two decades. Non-woven textiles used for filtering PPE are single-use products in the healthcare environment; use and protection is focused on preventing infection from airborne or aerosolized pathogens such as Influenza A virus or SARS-CoV-2. Recently, C–H bond activation under mild and controllable conditions was reported for crosslinking commodity aliphatic polymers such as polyethylene and polypropylene. Significantly, these are the same types of polymers used in PPE filtration systems. In this report, we take advantage of this C–H insertion method to covalently attach a photosensitizing zinc-porphyrin to the surface of a melt-blow non-woven textile filter material. With the photosensitizer covalently attached to the surface of the textile, illumination with visible light was expected to produce oxidizing ^1^O_2_/ROS at the surface of the material that would result in pathogen inactivation. The filter was tested for its ability to inactivate Influenza A virus, an enveloped RNA virus similar to SARS-CoV-2, over a period of four hours with illumination of high intensity visible light. The photosensitizer-functionalized polypropylene filter inactivated our model virus by 99.99% in comparison to a control.

## Introduction

The increase in global demand of filter materials for personal protective equipment (PPE) from the SARS-CoV-2 pandemic has highlighted the weakness of the global supply chain and presented the imminent possibility of a limited supply for individuals who require PPE to complete their job without an undue risk of (self-)infection^1,2^. Potential solutions have been explored to allow PPE reuse in circumstances where supply is not able to keep up with demand. Thus far, the main approaches have focused on treating contaminated materials to inactivate any pathogens captured by the filter. These solutions use available sterilization processes including heat, steam, ethylene oxide, hydrogen peroxide vapour, UV-C, microwaves, salt, and photosensitizers^3–8^. The research using currently available sterilization processes has focused on the number of cycles before device failure, the length of time for each sterilization cycle to be practical, and additional health risks—which for sterilization using ethylene oxide is currently in conflict^4,9^. Developing safe, reliable, and reusable filtering materials for PPE remains a challenge. Ideally, sterilizing should not require specialized equipment in order to improve access for healthcare workers and remote communities during times of global supply chain disruption and PPE shortages. In addition, there is potential to decrease the environmental impact of this large industry producing billions of single-use products per year that cannot be recycled. Finally, reports suggest contaminated surfaces and materials are able to produce aerosolized fomites (non-respiratory particles aerosolized from virus-contaminated surfaces) that are capable of spreading disease; specifically, it was hypothesized that the aerosolized fomites were originating during removal of contaminated PPE^10–12^.

An alternative approach to PPE reuse is to modify the base materials. Ideally, the materials possess filtering, continuous pathogen inactivation, and the ability to self-sterilize when exposed to a plentiful and accessible stimulus. Light is a stimulus that is abundant from both natural and synthetic sources, accessible, and relatively low in cost. Photodynamic therapy (PDT) is a technology that utilizes light (including within the visible spectrum) and photosensitizing (PS) molecules to eradicate pathogens, reduce infection, or eliminate unwanted or cancerous tissue through the production and reaction of singlet oxygen (^1^O_2_) and/or reactive oxygen species (ROS)^13,14^. PDT has been used extensively in vivo and can be used without causing undue bodily harm^14^. When illuminated with visible light the photosensitizer is excited and proceeds through an energy transfer process with abundant triplet oxygen (^3^O_2_) to produce ^1^O_2_ and ROS locally that can be used to eliminate pathogens. The activity is non-specific and can eliminate resistant pathogens that are typically difficult to eradicate^15–17^. As an added benefit, pathogens cannot evolve resistance to ^1^O_2_ as easily as they can to small molecule inhibitors^15,17^.

Polymer composites, natural and synthetic polymers, and silica particles have been covalently functionalized with photosensitizing molecules and used for microbial inactivation, with the majority of applications focusing on bacterial inactivation^18^. Non-covalent impregnation of photosensitizers into bulk polymer matrices has also been demonstrated to result in microbial inactivation^19^, but the lack of covalent attachment within these systems may lead to leaching of the photosensitizer into the environment. As a result, larger concentrations of the additive will be required in order to avoid a decrease in surface concentration—and therefore activity—over time. Furthermore, the leaching of active components can impact human and environmental health.

Covalent attachment approaches have the advantage of reducing the possibility of active component release (leaching) which would inevitably result in a decrease in performance over time. In addition, covalent attachment of photosensitizers can allow the surface of a material to be modified without affecting the bulk. This places the photosensitizer where it is needed (since only the surface of a material can interact with pathogens), thereby requiring less agent to achieve a desired response. In addition, the bulk properties of the substrate material (e.g. tensile strength or glass transition temperature) will be left unperturbed.

Rose Bengal, methylene blue, ruthenium-based complexes, pthalocyanines, and porphyrins have been covalently attached to natural and synthetic polymers that bear suitable chemical functionality to support the occurrence of chemical reactions at their surface^20–29^. These covalent attachment methods have been specifically designed for high-functionality materials that are not typically used in PPE/healthcare settings, or else require material pre-functionalization to allow ‘click chemistry’ (i.e. azide-alkyne coupling through Huisgen cycloadditions). A general solution for attaching photosensitizing molecules onto non-functional materials, such as aliphatic polymers used in PPE for example, remains an obstacle. These types of materials are comprised entirely of C–C and C–H bonds, and therefore lack any chemical “handles” that can be used for derivatization with photosensitizers.

One of our research groups recently described a family of *bis*-diazirine reagents that can be used to crosslink simple aliphatic polymers like polyethylene and polypropylene^30,31^. The reagents work by expelling nitrogen gas (N_2_) from the tethered diazirine groups upon thermal or photochemical stimulation, to afford high-energy carbenes which can then undergo rapid, low-barrier insertion into any available C–H bonds. In principle, such a technique can also be used to add functionality to base polymeric materials: the desired functional group can simply be tethered to one or more diazirine units, and the resulting conjugate can then be irreversibly linked to the surface of any polymeric material through appropriate activation of the diazirine function.

**Figure 1:**
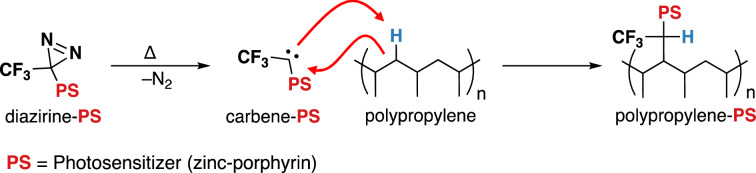
Strategy for creating covalently functionalized aliphatic polymer filters using diazirine C–H insertion chemistry and photosensitizers.

Herein, we employ this C–H activation strategy to permit the covalent functionalization of non-woven melt-blown polypropylene textiles with a common photoactive molecule (Fig. 1). The combination of different melt-blown polypropylene non-woven filters are what make up most surgical and N95 respirators; the cover layer, commonly called spun-bond polypropylene (**SBPP**) has a fiber density of ~ 15 g/m^2^; and the filter, commonly called, simply, melt-blown polypropylene (**MBPP**) has a density of ~ 30 g/m^2^. The lack of functional groups in this material means that it cannot be derivatized by traditional covalent linking methods. As a proof of concept, we demonstrated the inactivation of influenza A virus (IFV) using a visible light stimulus. IFV was chosen as a model because IFV itself is a major threat as a potential pandemic virus, and because IFV’s enveloped structure and RNA genome are similar to those of SARS-CoV-2—the organism that is the proximal cause of the current pandemic. Researchers have indicated previously the importance of developing and testing inactivation methods against enveloped viruses, given that a pandemic was expected long before the emergence of SARS-CoV-2^32^.

## Results and discussion

### Functionalization of non-woven polypropylene filter with photoactive porphyrin

**Figure 2:**
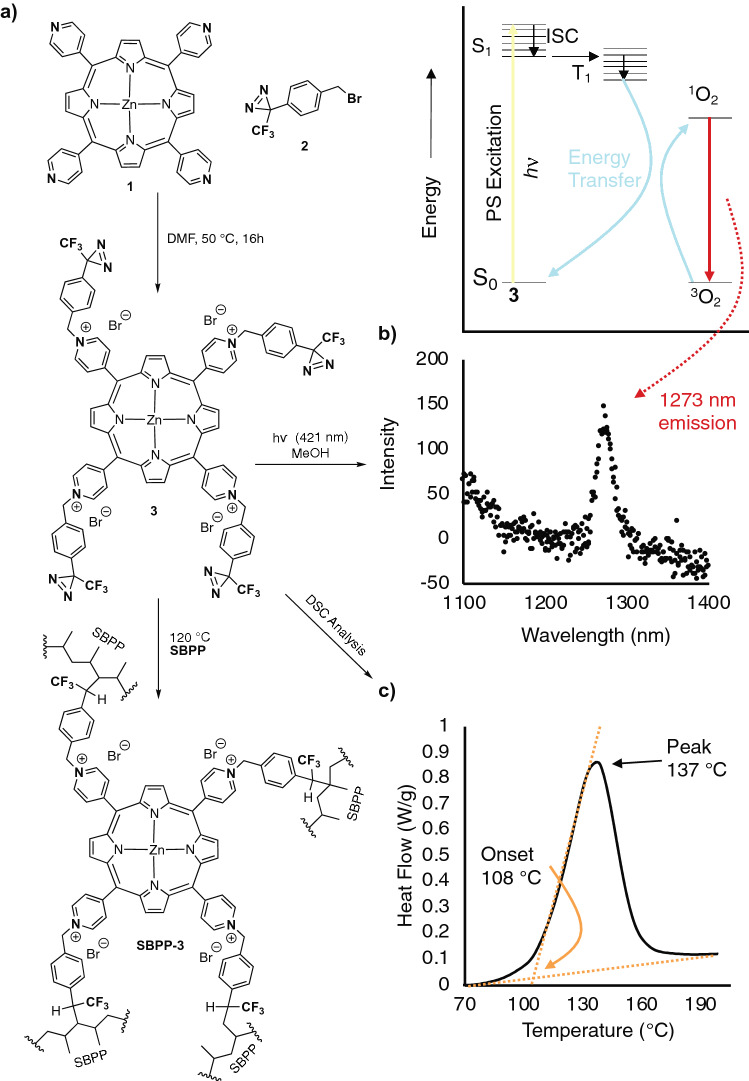
(**a**) Synthesis of **3** and subsequent **SBPP** functionalization. (**b**) ^1^O_2_ phosphorescence after excitation of **3** at 421 nm. (**c**) DSC of **3** indicating the onset and peak reaction temperature of the diazirine.

The **SBPP** and **MBPP** substrates were chosen because of the widespread use of the non-woven material in filtering PPE, specifically single-use surgical masks and N95/personal air purifying respirators. A commercially available photosensitizer (**1**) was selected, which possessed a high quantum yield for the production of singlet oxygen with visible light, and which possessed nucleophilic residues for easy derivatization. To facilitate insertion into the surface accessible C–H bonds of the **SBPP** and **MBPP**, we utilized a 3-trifluoromethyl-3-phenyl-3*H*-diazirine motif (TFPD) that is known to generate carbenes that are capable of ready insertion into C–H bonds^33^. A commercially available benzyl bromide (2) that incorporates the TFPD motif was reacted with the pyridyl groups of **1** to produce the desired tetrakis diazirinyl zinc-porphyrin **3** in near-quantitative yield (Fig. 2a). We confirmed ^1^O_2_ production with fluorescence spectroscopy from the excitation of **3** and subsequent phosphorescence of ^1^O_2_ at 1273 nm. The ^1^O_2_ generation proceeds through the photoexcitation of ground state **3** (S_0_) to generate the singlet excited state of **3** (S_1_), intersystem crossing to a triple state **3** (T_3_), and then an energy transfer to the triplet ground state of oxygen (^3^O_2_) to produce singlet oxygen (^1^O_2_) which then undergoes phosphorescence back to the ground state observed as an emission at a wavelength of 1273 nm indicating that the zinc-porphyrin moiety still possessed activity when functionalized with four diazirine groups (Fig. 2b).

The surface of the **SBPP** and **MBPP** were then covalently functionalized with **3** using a modified procedure from Lepage et al.^30^ To facilitate this, the reactivity of the diazirines in **3** was first analyzed by differential scanning calorimetry (DSC). These data revealed an onset temperature of 108 °C (determined by extrapolation of the tangent of the upward slope to the fitted baseline of the plot) and a peak temperature of 137 °C (Fig. 2c). With this information in hand, we chose to react compound **3** with **SBPP** and **MBPP** for 4 h at 120 °C (using 10 wt% of **3** with respect to **SBPP** or **MBPP**) to afford **SBPP-3** and **MBPP-3**, green-coloured textiles. The lower density textile **SBPP** (and subsequent functionalized **SBPP-3**) was used exclusively herein to test virus inactivation and quantify ROS/^1^O_2_ production. The higher density textile **MBPP** (and subsequent functionalized **MBPP-3**) was used to analyze the effect on filtration performance.

The intense green colour of **3** was advantageous for (1) ensuring coverage of **SBPP** (and **MBPP** in an analogous procedure) with the solution of **3**, and (2) monitoring the effectiveness of the diazirine C–H insertion reaction through the persistent green colour after washing (Fig. S5). The surfaces of **MBPP** in comparison to **MBPP-3** were imaged by scanning electron microscopy, which confirmed that there was no observable change to the surface of the fibers (Supplementary Fig. S6).

Prior to virus inactivation testing we completed a robust washing protocol analyzing the wash fractions with UV–Vis spectroscopy to observe the removal of **3** that did not covalently react with the surface of **SBPP**. The washing process was initially completed over ~ 6 days with 12 changes in solvent (Fig. S4). It was crucial to ensure complete removal of any **3** that did not covalently bind to **SBPP** during the C–H insertion step, to ensure that our subsequent virus inactivation testing focused on the abilities of **SBPP-3** exclusively. The efficacy of free antimicrobials is greater than that of an immobilized antimicrobial because of the ability to diffuse throughout a solution; in the case of an immobilized antimicrobial, the activity is reliant upon virus diffusion to within the proximity of the surface of the material where the interaction/reaction can occur. The lifetime of the ^1^O_2_/ROS produced (µs to ms) limits the space in which inactivation may take place, which we hypothesized would create a layer at and above the material’s surface that would inactivate any virus occupying that space^34,35^. The inactivation would then rely on pathogen diffusion in the transfer medium to within this concentrated ^1^O_2_ area on the **SBPP-3** surface and was therefore expected to present a time-dependence. Virus inactivation testing was then completed using **SBPP-3** using a minimal volume of carrier medium to mimic aerosolized and droplet capture by the filter.

IFV (strain A/California/07/2009) was used as a model enveloped RNA virus. IFV would provide insight into the inactivation performance of our material against enveloped RNA viruses similar to the SARS-CoV-2, which continues to cause a worldwide pandemic in 2021. We used a high-intensity LED light capable of producing ~ 30,000 lx to excite **SBPP-3** (see Supplementary Fig. 7 for manufacturer’s provided UV–Vis profile) which would mimic the amount of light that may be found in a hospital operating theatre or emergency area^36^.

**Figure 3:**
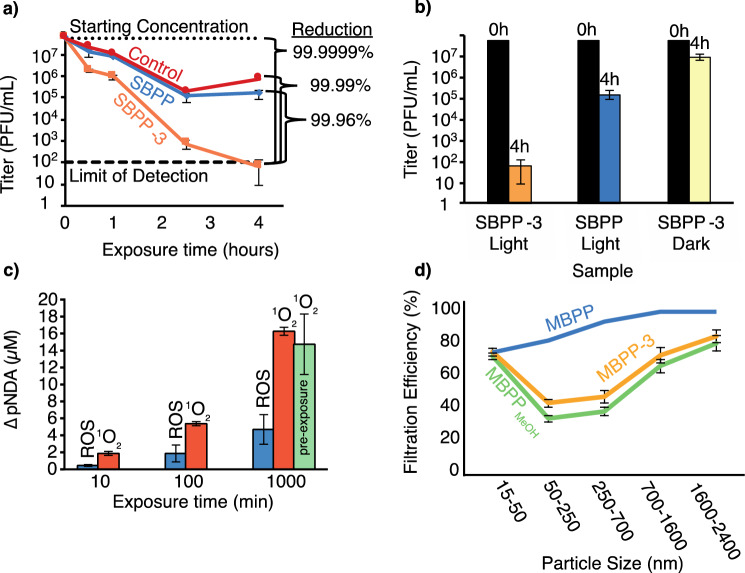
(**a**) Log_10_ Reduction of PFU/mL active virus vs. exposure time to visible light against the control (empty well), **SBPP**, and **SBPP-3**; (**b**) Log_10_ Reduction of PFU/mL active virus at 4 h against **SBPP-3** and **SBPP** with and without light in comparison to the starting virus concentration (0 h); (**c**) Relative change in scavenger pNDA concentration comparing quantities of ^1^O_2_ and ROS produced from 1 mg of **SBPP-3** during exposure to white light. Pre-exposure is an analogous ^1^O_2_ scavenging experiment with **SPBB-3** after a exposure to 30,000 lx of white light for 4 days. A larger value of ΔpNDA indicates more of that species produced; (**d**) Filtration efficiency of **MBPP**, **MBPP**_**MeOH**_, and **MBPP-3**.

The virus particles were exposed to the light for between 0.5 and 4 h in 10 µL of tissue culture medium in a well of a 96-well plate that contained a test surface (see Supplementary Fig. S5). After 1 h exposure the virus titer was reduced by 1.06 log with **SBPP-3**, compared with only a minor 0.13 log reduction on the **SBPP** control surface (Fig. 3a). Over the 4-h testing period a logarithmic relationship between reduction of PFUs and exposure time was observed. At 4 h of exposure, the treated surface resulted in a 4.07 log reduction (99.99%) in comparison to an empty well, a 3.38 log reduction (99.96%) in comparison to untreated **SBPP** and a 5.95 log reduction (99.9999%) in comparison to the starting virus PFU concentration.

These data indicate that near-sterilization levels of inactivation may be achievable over longer periods (4 + hours) of high-intensity illumination of **SBPP-3**, offering an alternative approach to re-sterilization and re-use of PPE. A distinct advantage of chemically modifying the material is to facilitate continuous sterilization during use, which would decrease the potential for aerosolized fomites when using or removing PPE, thereby decreasing a potential mode of infection^10^.

To differentiate the impact that immobilized **3** and visible light contribute to the viral inactivation efficacy, we completed an analogous virus exposure test of **SBPP-3** in the absence of light (Fig. 3b). Without exposure to light, **SBPP-3** minimally inactivated the inactivated the virus with a 0.76 log reduction (Fig. 3b; **SBPP-3 Dark**), which was substantially less than **SBPP** exposed to light. These data indicated that immobilized **3** and visible light are required to achieve efficient virus inactivation, and that switchable on/off performance and lithography/patterning of virus inactivation may be possible with this system.

To confirm the presence and quantity of the sensitized molecular species—^1^O_2_ and ROS—responsible for inactivating the viruses upon exposure to white light, a selective scavenging experiment utilizing *p*-nitrosoaniline (pNDA) was employed (see Method section for details)^38^. pNDA bleaching by ROS can be quantified spectrophotometrically by the reduction of absorbance of pNDA at 440 nm ; comparatively, ^1^O_2_ is not capable of bleaching pNDA directly. Utilizing a combination of pNDA and imidazole—with the imidazole capable of reacting efficiently with ^1^O_2_ to produce an intermediate species capable of bleaching pNDA—the quantification of ^1^O_2_ can then be determined through the reduction of the absorbance of pNDA at 440 nm in comparison to the ROS specific experiment above. White light exposure of **SBPP-3** in combination with pNDA (scavenging ROS) or pNDA + imidazole (scavenging ROS and ^1^O_2_) for a duration of 10, 100, and 1000 min was completed and analyzed by UV–Vis spectroscopy at 440 nm and plotted as a change in concentration of pNDA vs. exposure time (Fig. 3c). The results indicated that in the presence of light **SBPP-3** primarily generates ^1^O_2_ and not other ROS. Additionally, to probe the stability of the photosensitizer over longer periods of white light exposure, a sample of **SBPP-3** was exposed to high intensity white light for 4 days, and then underwent an analogous 1000-min ^1^O_2_ quantification experiment (white light + pNDA + imidazole); the results indicated the material had a similar level of ^1^O_2_ production, albeit with a larger standard deviation (Fig. 3c, labelled as pre-exposure at 1000 min).

Lastly, the effect of functionalization on filtration efficiency was tested using **MBPP-3**; **SBPP** materials are typically the outer and inner coverings on masks and do not possess a high degree of filtration (below 10% filtration efficiencies below 2 µm particles) whereas **MBPP** materials have a higher density of thinner diameter fibers that enable higher filtration efficiencies with multiple layers. Filtration efficiency of pristine **MBPP** was compared to **MBPP-3** and **MBPP** that underwent an analogous procedure, exclusive of additive **3**—denoted **MBPP**_**MeOH**_. The **MBPP-3** and **MBPP**_**MeOH**_ had equivalent filtration efficiencies, indicating that the photosensitizer **3** had no negative effect on filtration performance (Fig. 3d). The decrease in filtration performance of **MBPP**_**MeOH**_ and **MBPP-3** in comparison to **MBPP** was caused by the elimination of the electrostatic charge; methods to regenerate the electrostatic charge on the filters have been explored by others with success and provides a possible method if post-production functionalization is utilized^37^.

The ability to functionalize aliphatic polymers with diazirines is a relatively new methodology and has the potential to impact a large portion of the commodity polymer market. Low-functionality aliphatic polymers—such as the SBPP/MBPP used in this research—account for the largest three (polypropylene, low-density and high-density polyethylene) volumes of thermoplastics in the world equating to ~ 36% or 23 million tons of polymers used worldwide each year^34^. With over 39% in packaging and 22% in other areas that include health and safety, the potential for improvement in product performance and safety across different markets that utilize aliphatic polymers is high. Furthermore, this method of functionalization can be applied to different commodity polymers including polystyrene, polyurethane, polyethylene terephthalate, nylon, and acrylic and as such may allow recycled materials an avenue for use in high-performance and advanced material applications from renewable resources^39^. The diazirine functionality is not limited to C–H bond activation and can also be used to functionalize more reactive O–H and N–H bonds in polyalcohols and polyamides which could be used for post-processing functionalization of different materials that would benefit from self-sterilization in healthcare settings^30^.

Future testing will focus on the development of this technology to understand the performance limitations of the material with respect to photosensitizer loading and surface coverage, repeated sequential virus inactivation, potential photobleaching, light intensity-dependent performance, response to washing and detergents, and performance against different types of pathogens. The optimization of inactivation materials should be focused on developing technology that is broadly applicable, robust, and possesses broad spectrum activity without the potential for developing resistance.

## Conclusions

We have described the production of a polypropylene-based non-woven filter that was covalently functionalized with a zinc-porphyrin photosensitizer using diazirine C–H activation chemistry. The **MBPP-3** material was tested against a model virus, Influenza A, to explore the virus inactivation abilities of the material when exposed to visible light. Over a 4-h incubation period with exposure to visible light there was 4-log reduction of active virus resulting in a logarithmic trend in the inactivation of virus over time, and a 5.95 log reduction in comparison to the starting virus titer. This research presents a new approach to achieving functionalized aliphatic polymers, such as those used in PPE, which provides a potential solution for reducing pathogen transfer; a route for designing re-useable and re-sterilizable PPE that can reduce the need for single-use products; and the development of post-functionalization processes that could see widespread use across many different forms of PPE.

## Methods

All chemicals were used as received. Zinc 5,10,15,20-*tetra*(4-pyridyl)-21*H*,23*H*-porphine was purchased from Sigma Aldrich (Missouri, USA). 4-[3-(Trifluoromethyl)-3*H*-diazirin-3-yl]benzyl bromide was purchased from TCI America (Portland, USA). Spunbond polypropylene (SBPP, 15 g/m^2^) was supplied by Oxco, Inc (Fort Mill, South Carolina). Melt-blown polypropylene (MBPP, 30 g/m^2^) was removed from surgical masks (EN14683: 2019 Type IIR, Asia Dynamics Inc, Zurich). EM-X090 LED light with incorporated driver (90 W) was purchased from Jons Plant Factory (Burnaby, Canada) and used for virus inactivation experiments and a Venso Duo LED light purchased from Galaxus (Switzerland) for ^1^O_2_/ROS quantification experiments. Illuminance (luminous flux/unit area, lux = lumens/m^2^) was measured with the Lux Light Meter app using an Apple iPhone. For experimental virus inactivation and LED light setup see Supplementary Fig. 5. Fluorescence spectroscopy measurements were completed on a Horiba Jobin Yvon Fluorolog‐3 fluorimeter equipped with an Xe arc lamp and a TBX single‐photon counter in a quartz cuvette. Scanning Electron Microscopy was completed using an FEI Quanta 200F with EDAX Octane Super. UV–Vis spectroscopy was completed using a BioTek Epoch Microplate Spectrophotometer. Filtration efficiency tests were completed using circular test specimens with a diameter of 4.6 cm (sample diameter 6 cm). An aerosol consisting of neutralized sugar particles of 15 to 2400 nm diameter and a concentration in air of approx. 35 mg/m^3^ was led over the test specimen. A constant air flow of of 8 l/min (8 cm/s) is generated by the aid of a vacuum through the specimen based on DIN EN 14,683. The particles diffusing through the specimen are quantified in real-time by use of a particle analyzer (Cambustion DMS500). The particle filtration efficiency is given in % and determined after achieving a steady-state flow of particles (after ~ 2 min) by comparison of the particle flow through the filter system with the raw aerosol. Results are reported in particle ranges of 15–50 nm, 50–250 nm, 250–700 nm, 700–1600 nm and 1600–2400 nm.

### Synthesis of tetra diazirine zinc porphyrin (3)

Zinc 5,10,15,20-*tetra*(4-pyridyl)-21*H*,23*H*-porphine (ZnTPyP, **1**) (70 mg, 0.102 mmol) was dissolved in 2 mL of DMF, then 4-[3-(trifluoromethyl)-3*H*-diazirin-3-yl]benzyl bromide (**2**) (171 mg, 0.61 mmol) was added to the solution and the reaction was heated to 50 °C and stirred vigorously for 16 h. The solvent was then evaporated under reduced pressure and the crude product **3** (211 mg, 0.10 mmol, 99%) was obtained as dark green solid. ^1^H NMR (300 MHz, MeOD) δ 9.49 (d, *J* = 6.6 Hz, 8H), 9.15 (s, 8H), 8.95 (d, *J* = 6.7 Hz, 8H), 8.01 (d, *J* = 8.5 Hz, 8H), 7.55 (d, *J* = 7.9 Hz, 8H), 6.31 (s, 8H). ^13^C NMR (126 MHz, MeOD) δ 164.88, 161.79, 150.37, 144.22, 136.60, 134.69, 134.03, 131.81, 131.62, 130.87, 129.74, 128.94, 128.38, 123.50 (q, *J* = 273.9 Hz), 117.30, 64.82, 37.00, 35.40, 32.55, 31.68, 29.45 (q, *J* = 40.7 Hz). ^19^F NMR (283 MHz, MeOD) δ–66.90.

### Production of zinc porphyrin functionalized spunbond and meltblown polypropylene (SBPP-3 and MBPP-3, respectively)

**SBPP** or **MBPP** was cut into 4 cm diameter circles (with an initial white colour) and placed in 4 cm diameter aluminum weigh boats. Each filter piece was submerged in a methanol solution of **3** (equating to a concentration of **3** that was 10 wt% of the **SBPP** or **MBPP** textile) and was subsequently covered with aluminum foil and allowed to incubate at room temperature for 1 h. The aluminum pans containing the **SBPP** or **MBPP** and **3** were then uncovered and the methanol solvent was allowed to evaporate in the dark for 4 h which resulted in a green coloured **SBPP** or **MBPP** textile. The pans containing **SBPP** or **MBPP** and **3** were then incubated for 4 h at 120 °C in an oven. The resulting **SBPP-3** and **MBPP-3** textile was then washed with ethanol to remove any residual non-bound **3** by incubating the textile in 10 mL (first 10 washings) then 500 mL (last two washings) of ethanol until no remaining **3** was observed by UV–Vis spectroscopy. The **SBPP-3** and **MBPP-3** textiles were then air dried and used for virus inactivation testing, ^1^O_2_/ROS quantification, and filtration efficiency testing.

### Virus inactivation testing

**SBPP-3** was punched to precisely fit the bottom of a 96-well plate, soaked in 70% ethanol for 10 min, and air dried prior to testing. **SBPP** and **SBPP-3** were tested with 9 replicates and controls (empty wells) were completed in triplicate. Influenza A virus (A/California/07/2009 (H1N1)pdm09 virus, International Reagent Resources) in Dulbecco’s Modified Eagle medium (DMEM, 10 µL) was added to each well at a titer of 5.98 × 10^7^ plaque forming unit (PFU)/mL. The plate was exposed for the indicated time to visible light (31,951–30,198 lx at the 96-well plate) or wrapped with aluminum foil for the dark/no light exposure experiment) with a temperature range between 24 (start) to 30.2 ℃ (end). Following exposure, 50 µL phosphate buffered saline (PBS) was added to resuspend remaining viruses from the wells and pooled in triplicates, resulting in 3 samples each from **SBPP-3** and **SBPP** containing wells, and 1 sample for negative control. The samples were split into aliquots and stored at − 80 ℃ until use. Virus titers were determined by plaque assay. In brief, virus samples were serially diluted tenfold in DMEM. Madin-Darby Canine Kidney cell monolayers (ATCC) in 6-well plates were inoculated with 200 µL of virus at each dilution in triplicate. After 1 h adsorption at 37 ℃, the inoculum was removed and the monolayers were overlaid with DMEM containing 1% Noble Agar and 0.0075% trypsin. After incubation at 37 ℃ for 48 h, plaques were stained with neutral red and counted. Antiviral efficacy was calculated by comparing the number of resultant plaques.

### ^1^O_2_ and ROS scavenging experiment

The procedure was adapted from Kraljic and El Mohsni^38^. ^1^O_2_* Quantification:* 300 µL of a solution containing Dimethyl-4-nitrosoaniline (paranitrosodimethylaniline, 25 µM) and imidazole (925 µM) in 0.01 M phosphate buffer solution (pH = 7) was combined with a 1 mg sample of **SBPP-3** in a 96-well plate and irradiated with 30,000 lx of white light for 10, 100, and 1000 min. At each time segment, the **SBPP-3** samples were removed and the absorption of the pNDA/IM solution was obtained at 440 nm. For the 4 day pre-exposure sample, **SBPP-3** was exposed to 30,000 lx of white light in the dry state for 4 days, and then underwent an analogous procedure for ^1^O_2_ scavenging over 1000 min. The change in concentration of pNDA was calculated by using a standard curve of pNDA in a 0.01 M phosphate buffer solution. *ROS Quantification:* 300 µL of a solution containing Dimethyl-4-nitrosoaniline (paranitrosodimethylaniline, 25 µM) in 0.01 M phosphate buffer solution (pH = 7) was combined with a 1 mg sample of **SBPP-3** in a 96-well plate and irradiated with 30,000 lx of white light for 10, 100, and 1000 min. At each time segment, the **SBPP-3** samples were removed and the absorption of the pNDA solution was obtained at 440 nm. The change in concentration of pNDA was calculated by using a standard curve of pNDA in a 0.01 M phosphate buffer solution.

## Supplementary Information

Supplementary Information.
