# Supplementary material for: Covalent functionalization of polypropylene filters with diazirine–photosensitizer conjugates producing visible light driven virus inactivating materials﻿
Source: Sci Rep. 2021 Sep 24;11:19029. doi: 10.1038/s41598-021-98280-6 (PMC8463589; doi:10.1038/s41598-021-98280-6)
Supplement: Supplementary file 1 — Supplementary Information. [file 41598_2021_98280_MOESM1_ESM.docx]

**Supplementary Information**

**Covalent Functionalization of Melt-Blown Polypropylene Filters with Diazirine-Photosensitizers Producing Visible Light Driven Virus Inactivating Materials**

*Authors*: T.J. Cuthbert*^,1,2^, S. Ennis^3^, S. F. Musolino^4^, H. L. Buckley^5^, M. Niikura^3^, J.E. Wulff^4^, C. Menon^1,2^

Address:^1^ Department of Health Sciences and Technology, ETH Zürich, Zürich, 8008, Switzerland; ^2^ Schools of Mechatronic Systems Engineering & Engineering Science, Simon Fraser University, Metro Vancouver, BC V5A 1S6, Canada; ^3^ Faculty of Health Sciences, Simon Fraser University, Burnaby, BC V5A 1S6, Canada ^4^ Department of Chemistry, University of Victoria, Victoria, BC, V8W 3V6, Canada; ^5^ Department of Civil Engineering, University of Victoria, BC, V8W 3V6, Canada

*Email: tyler.cuthbert@hest.ethz.ch

Supplementary Figure 1. ^1^H NMR Spectrum of **3**.

Supplementary Figure 2. ^13^C NMR Spectrum of **3.**

Supplementary Figure 3. ^19^F NMR Spectrum of **3.**

Supplementary Figure 4. Leaching Experiment UV-Vis Spectrum of 6 days.

Supplementary Figure 5. Virus inactivation test setup with LED high intensity light and close up of **SBPP**, an empty well (control), and **SBPP-3**

Supplementary Figure 6. Low Pressure Scanning Electron Images of the surfaces of **MBPP**, and **MBPP-3.**


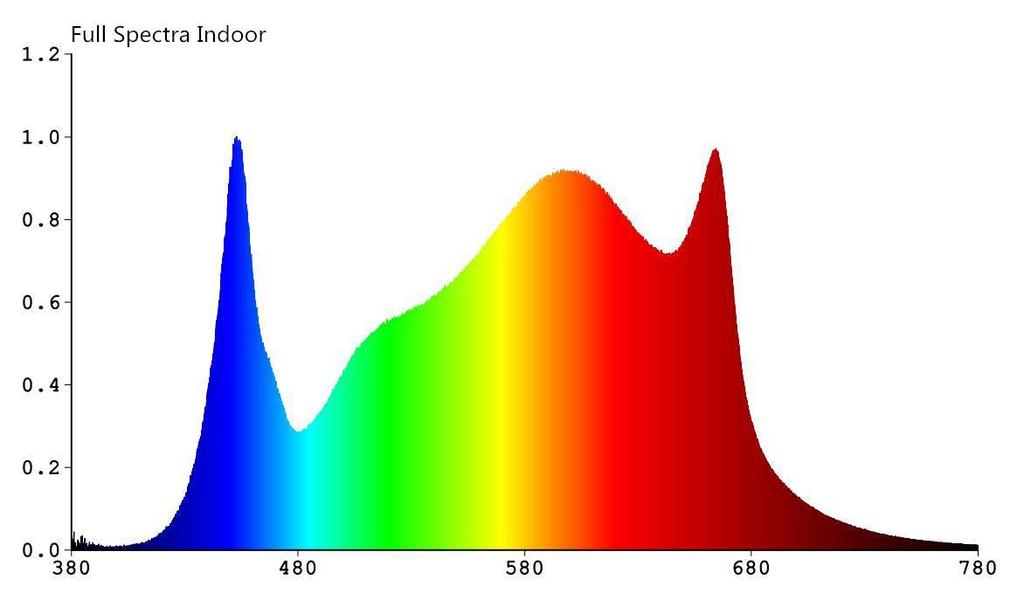


Supplementary Figure 7. EM-X090 LED UV-Vis output intensity spectrum.
